# *PCSK9* loss of function is protective against extra-coronary atherosclerotic cardiovascular disease in a large multi-ethnic cohort

**DOI:** 10.1371/journal.pone.0239752

**Published:** 2020-11-09

**Authors:** Aeron M. Small, Jennifer E. Huffman, Derek Klarin, Julie A. Lynch, Themistocles Assimes, Scott DuVall, Yan V. Sun, Labiba Shere, Pradeep Natarajan, Michael Gaziano, Daniel J. Rader, Peter W. F. Wilson, Philip S. Tsao, Kyong-Mi Chang, Kelly Cho, Christopher J. O’Donnell, Juan P. Casas, Scott M. Damrauer, on behalf of the VA Million Veteran Program

**Affiliations:** 1 Corporal Michael J. Crescenz VA Medical Center, Philadelphia, PA, United States of America; 2 Department of Medicine, Yale University School of Medicine, New Haven, CT, United States of America; 3 Massachusetts Veterans Epidemiology Research and Information Center (MAVERIC), VA Boston Healthcare System, Boston, MA, United States of America; 4 Boston VA Healthcare System, Boston, MA, United States of America; 5 Center for Genomic Medicine, Massachusetts General Hospital, Harvard Medical School, Boston, MA, United States of America; 6 Program in Medical and Population Genetics, Broad Institute of MIT and Harvard, Cambridge, MA, United States of America; 7 Department of Surgery, Massachusetts General Hospital, Boston, MA, United States of America; 8 Department of Veterans Affairs, Salt Lake City Health Care System, Salt Lake City, UT, United States of America; 9 University of Massachusetts College of Nursing & Health Sciences, Boston, MA, United States of America; 10 Center for Healthcare Organization and Implementation Research, Edith Nourse Rogers Memorial VA Hospital, Bedford, MA, United States of America; 11 Department of Medicine, VA Palo Alto Health Care System, Palo Alto, CA, United States of America; 12 Stanford University School of Medicine, Stanford, CA, United States of America; 13 Veterans Affairs Informatics and Computing Infrastructure; 14 Atlanta VA Health Care System, Decatur, GA, United States of America; 15 Department of Epidemiology, Emory University Rollins School of Public Health, Atlanta, GA, United States of America; 16 Department of Biomedical Informatics, Emory University School of Medicine, Atlanta, GA, Unites States of America; 17 Palo Alto Epidemiology Research and Information Center for Genomics, VA Palo Alto Health Care System; 18 Cardiovascular Research Center, Massachusetts General Hospital, Boston, MA, United States of America; 19 Department of Medicine, Brigham and Women’s Hospital, Harvard Medical School, Boston, MA, United States of America; 20 Department of Medicine, Perlman School of Medicine, University of Pennsylvania, Philadelphia, PA, United States of America; 21 Emory Clinical Cardiovascular Research Institute, Atlanta, GA, United States of America; 22 Cardiovascular Medicine Division, Department of Medicine, Brigham and Women’s Hospital, Harvard Medical School, Boston, MA, United States of America; 23 Department of Surgery, Perelman School of Medicine, University of Pennsylvania, Philadelphia, PA, United States of America; Stellenbosch University Faculty of Medicine and Health Sciences, SOUTH AFRICA

## Abstract

**Background:**

Therapeutic inhibition of PCSK9 protects against coronary artery disease (CAD) and ischemic stroke (IS). The impact on other diseases remains less well characterized.

**Methods:**

We created a genetic risk score (GRS) for *PCSK9* using four single nucleotide polymorphisms (SNPs) at or near the *PCSK9* locus known to impact lower LDL-Cholesterol (LDL-C): rs11583680, rs11591147, rs2479409, and rs11206510. We then used our GRS to calculate weighted odds ratios reflecting the impact of a genetically determined 10 mg/dL decrease in LDL-C on several pre-specified phenotypes including CAD, IS, peripheral artery disease (PAD), abdominal aortic aneurysm (AAA), type 2 diabetes, dementia, chronic obstructive pulmonary disease, and cancer. Finally, we used our weighted GRS to perform a phenome-wide association study.

**Results:**

Genetic and electronic health record data that passed quality control was available in 312,097 individuals, (227,490 White participants, 58,907 Black participants, and 25,700 Hispanic participants). *PCSK9* mediated reduction in LDL-C was associated with a reduced risk of CAD and AAA in trans-ethnic meta-analysis (CAD OR 0.83 [95% CI 0.80–0.87], p = 6.0 x 10^−21^; AAA OR 0.76 [95% CI 0.68–0.86], p = 2.9 x 10^−06^). Significant protective effects were noted for PAD in White individuals (OR 0.83 [95% CI 0.71–0.97], p = 2.3 x 10^−04^) but not in other genetic ancestries. Genetically reduced PCSK9 function associated with a reduced risk of dementia in trans-ethnic meta-analysis (OR 0.86 [95% CI 0.78–0.93], p = 5.0 x 10^−04^).

**Conclusions:**

Genetically reduced PCSK9 function results in a reduction in risk of several important extra-coronary atherosclerotic phenotypes in addition to known effects on CAD and IS, including PAD and AAA. We also highlight a novel reduction in risk of dementia, supporting a well-recognized vascular component to cognitive impairment and an opportunity for therapeutic repositioning.

## Introduction

The discovery of loss-of-function (LoF) variants in *PCSK9* provided strong evidence that therapeutic manipulation of PCSK9 can prevent coronary artery disease (CAD) through reduction of low-density lipoprotein cholesterol (LDL-C) [1]. This functional hypothesis has been confirmed by the reduction in CAD and ischemic stroke (IS) reported with PCSK9 inhibition in randomized trials [2, 3]. Evaluation of the association between genetic variation in *PCSK9* and diverse outcomes provides the opportunity to identify unexpected effects of PCSK9 inhibition that are not likely to be evident during the short follow up time of clinical trials. Notably, there are several such reports demonstrating an increased risk of type 2 diabetes (T2D) for individuals with *PCSK9* LoF [4–6].

In this study we leveraged the large size and diversity of the VA Million Veteran Program (MVP) to identify the phenotypic consequences of genetic variation in *PCSK9* function. We specifically considered CAD, peripheral artery disease (PAD), ischemic stroke (IS), and abdominal aortic aneurysm (AAA), as well as pre-specified non-atherosclerotic diseases, including T2D, dementia, chronic obstructive pulmonary disease (COPD), and cancer, which are common in the clinical population likely to be treated with PCSK9 inhibitors. We subsequently performed a phenome wide association study (PheWAS) in order to identify additional associated phenotypes.

## Materials and methods

This study was approved by the Department of Veteran’s Affairs Central Institutional Review Board.

All phenotypes were determined as of enrollment in the MVP. Atherosclerotic phenotypes were developed from a combination of diagnosis and procedure codes present in the VA electronic health record (EHR) of MVP participants (S1 Table in S1 File). Pre-selected non-atherosclerotic phenotypes were developed from a combination of appropriate diagnostic billing codes (S2 Table in S1 File). Phenotyping for PheWAS was performed using the phecode method, described elsewhere [7]. Lipid phenotypes represent maximum (LDL-C, total cholesterol, triglycerides) or minimum (HDL-C) values present in the EHR at any time prior to enrollment. Statin use was adjudicated for all MVP participants as of enrollment.

We created a genetic risk score (GRS) for *PCSK9* function using four single nucleotide polymorphisms (SNPs) at or near the *PCSK9* locus that have been previously demonstrated in a genetic risk score to strongly associate with lower LDL-C in individuals of European genetic ancestry [4]: rs11583680, rs11591147, rs2479409, and rs11206510.

Using participant level data, we combined these SNPs in a gene-centric score weighted by each variant’s effect on LDL-C within MVP [8] and calculated the association of a one-standard deviation this score with lipid traits (total cholesterol, LDL-C, HDL-C, and triglycerides), controlling for age, sex, and 5 ancestry-specific principal components using linear regression. We then tested the association of a one standard deviation change in the gene-centric score with pre-specified atherosclerotic and non-atherosclerotic traits. Using these results we then calculated adjusted odds ratios to reflect the odds that a phenotype (outcome) occurs given a genetic risk score burden equivalent to a 10 mg/dL difference in LDL-C from the population mean (exposure). We considered a Bonferroni corrected p-value of 6.3x10^-3^ (p = 0.05/n = 8) significant. Statin use was added as a covariate for sensitivity analysis for all atherosclerotic phenotypes and for select other phenotypes meeting our significance threshold in the primary analysis.

PheWAS was performed using the PheWAS package in R [9]. For PheWAS, we limited our analysis to traits which had greater than 200 cases in all three genetic ancestries (n = 507) and used a Bonferroni corrected p-value of 9.x10^-5^ (p = 0.05/n = 507) as a significance threshold. All PheWAS analyses were adjusted for age, sex, and 5 ancestry-specific principal components. Ancestry specific PheWAS were meta-analyzed using inverse variance weighted meta-analysis.

## Results

**Fig 1:**
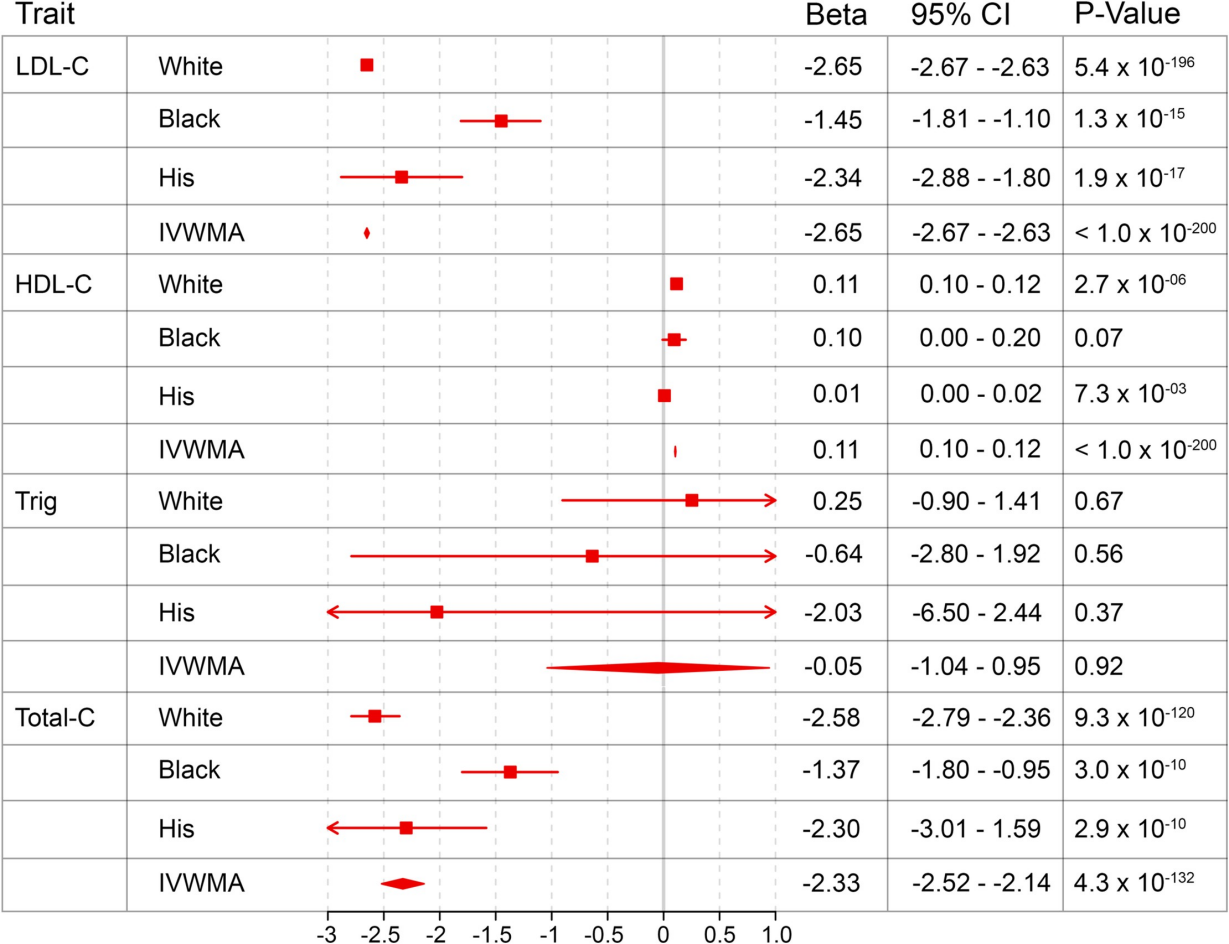
Effect of 1 standard deviation difference in *PSCK9* genetic risk score on lipid traits in mg/dL. Beta and 95% confidence interval is displayed for the effect of our *PCSK9* genetic risk score on lipid traits for White individuals, Black individuals, Hispanic individuals, and inverse variance weighted meta-analysis of White, Black, and Hispanic populations (IVWMA). All analyses were performed controlling for age, sex, and 5 ancestry-specific principal components.

Genetic and EHR data passing quality control were available in 312,097 individuals [8] including 227,490 (72.9%) White participants, 58,907 (18.9%) Black participants, and 25,700 (8.2%) Hispanic participants. Minor allele frequencies by genetic ancestry for each SNP in the GRS are described in S3 Table in S1 File (S3 Table in S1 File). For every standard deviation change in GRS, we observed a reduction in LDL-C of 2.65 mg/dL, 1.45 mg/dL, and 2.34 mg/dL in White, Black, and Hispanic individuals, respectively (Fig 1).

**Fig 2:**
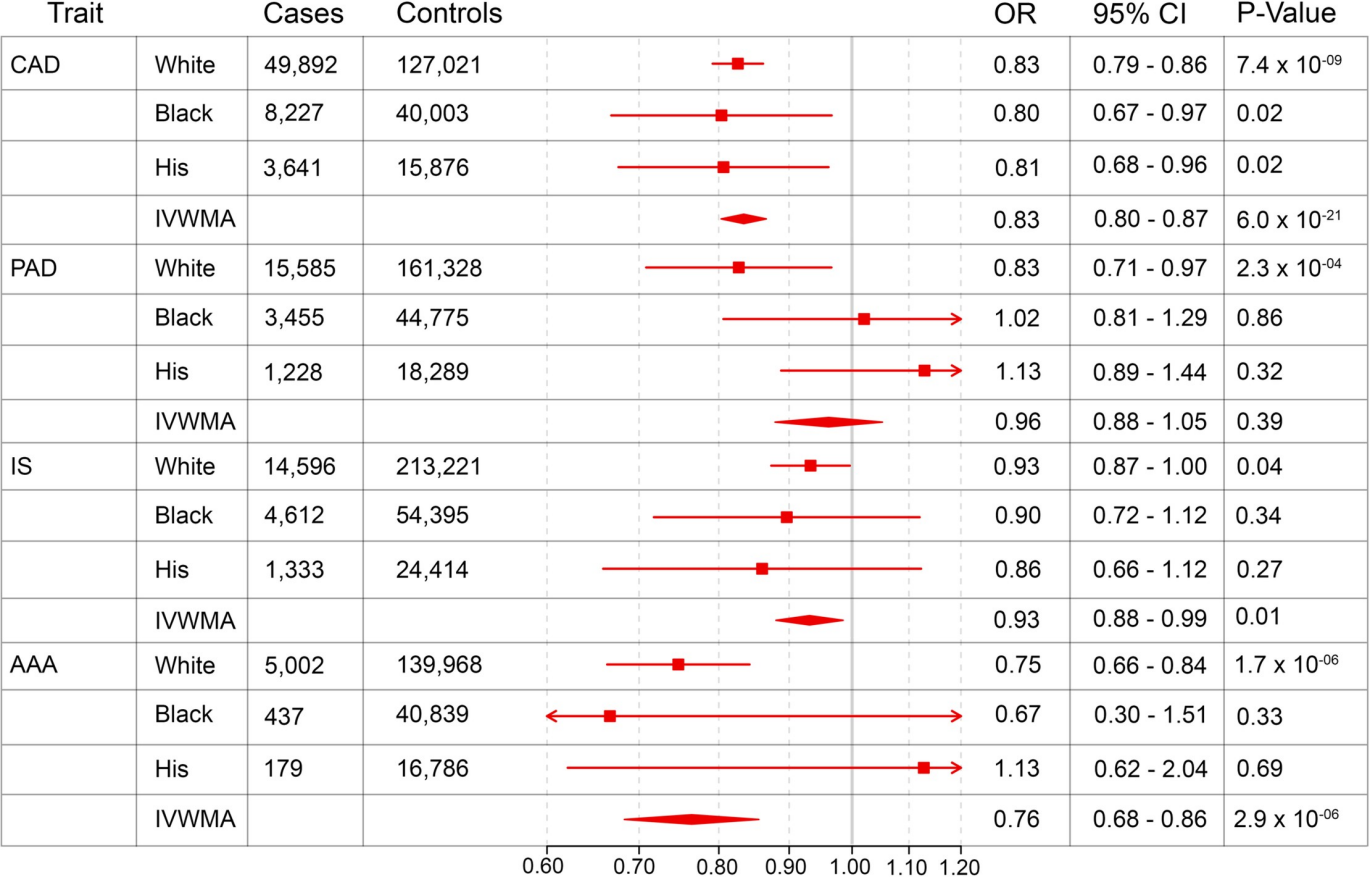
Weighted odds ratios reflecting the impact of a genetically determined 10 mg/dL decrease in LDL-C on pre-specified atherosclerotic traits. Odds Ratio and 95% confidence interval is displayed for the effect of a genetically determined 10 mg/dL decrease in LDL-C on primary atherosclerotic traits for White individuals, Black individuals, Hispanic individuals, and inverse variance weighted meta-analysis of White, Black, and Hispanic populations (IVWMA). All analyses were performed controlling for age, sex, and 5 ancestry-specific principal components.

**Fig 3:**
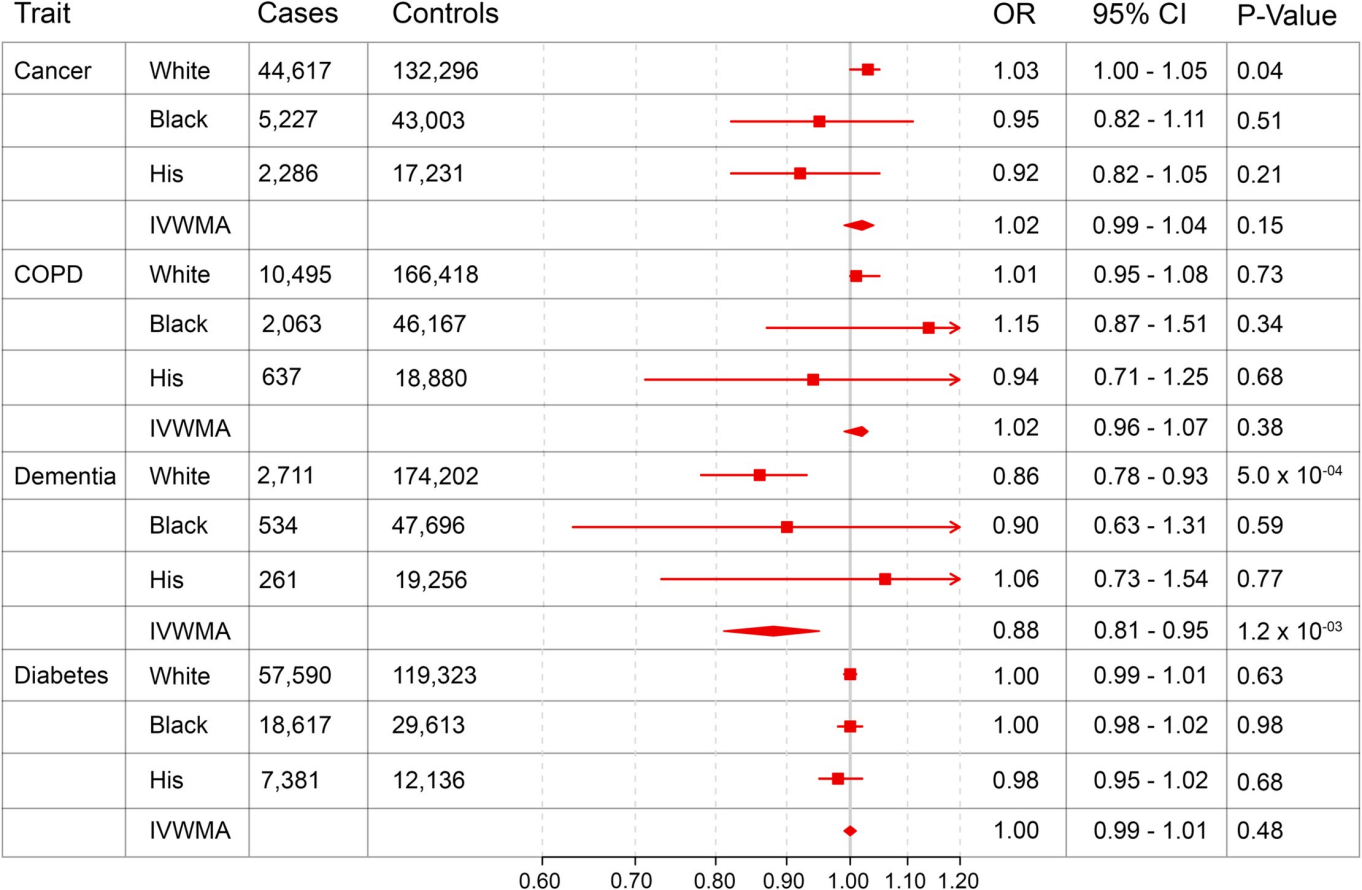
Weighted odds ratios reflecting the impact of a genetically determined 10 mg/dL decrease in LDL-C on safety and efficacy endpoints. Odds ratio and 95% confidence interval is displayed for the effect of a genetically determined 10 mg/dL decrease in LDL-C on safety and efficacy endpoints for White individuals, Black individuals, Hispanic individuals, and inverse variance weighted meta-analysis of White, Black, and Hispanic populations (IVWMA). All analyses were performed controlling for age, sex, and 5 ancestry-specific principal components.

Fig 2 summarizes the effect of the genetic variation in *PSCK9* on atherosclerotic diseases and Fig 3 summarizes the same for pre-selected non-atherosclerotic endpoints. PCSK9 mediated reductions in LDL-C were associated with an approximately 20% reduced risk of both CAD and AAA in trans-ethnic meta-analysis. Significant protective effects were noted for PAD in White individuals, but not in other genetic ancestries or in trans-ethnic meta-analysis. A nominal, but not experiment-wide, significant association was seen between *PCSK9* mediated LDL-C reduction and IS in White individuals and in meta-analysis. Of our non-atherosclerotic endpoints, genetically determined reduced *PCSK9* function was associated with a reduced risk of dementia in trans-ethnic meta-analysis. In contrast to previous reports, there was no evidence for increased risk of T2D associated with genetically diminished *PCSK9* function.

The addition of statin use as a covariate attenuated the strength of association between our GRS and all selected phenotypes. Of the three phenotypes meeting our pre-specified Bonferroni correction in primary analysis (CAD, AAA, and dementia), only CAD and AAA maintained a significant association with our GRS after adjusting for statin use, though both the effect estimates and significance were diminished (S4 Table in S1 File).

A total of 11 traits reached our significance threshold in trans-ancestry PheWAS including traits related to dyslipidemia (hyperlipidemia, disorders of lipid metabolism, hypercholesterolemia, mixed hyperlipidemia), coronary disease (coronary atherosclerosis, ischemic heart disease, other chronic ischemic heart disease, angina pectoris, and unstable angina), and arterial aneurysms (other aneurysm, aortic aneurysm) (S5 Table in S1 File).

## Discussion

We generated a GRS predicting *PCSK9* function and calculated its impact on risk for a variety of atherosclerotic and non-atherosclerotic phenotypes within the MVP. Our GRS confirm and extend evidence that reduction in LDL-C reduces risk for CAD and IS, observed in previous genetic studies and in clinical trials of PCSK9 inhibitors. We also demonstrated that genetic reduction in *PCSK9* function is associated with a reduced risk of other atherosclerotic diseases, including PAD, and AAA. Lastly, we demonstrate a novel association between genetically determined reduction in PCSK9 function and protection against dementia.

AAA is prevalent in 4–8% of the population overall, and is more common in the elderly and in males [10, 11]. We demonstrated that genetic reduction in *PCSK9* function significantly reduces the risk of AAA, a finding that was previously described in PheWAS of the rs11591147 variant and Mendelian randomization experiments in MVP [8]. This result is further supported by a meta-analysis of independent experiments using GRSs of lipid trait-associated SNPs to evaluate AAA risk [12]. Taken together, these data highlight a potential role for LDL-C modulating therapies such as PCSK9 inhibition in preventing AAA and identifies avenues for future clinical trials.

Our data further demonstrated that within White individuals, *PCSK9* inhibition reduced the risk of atherosclerotic PAD. This finding is consistent with results from the Further Cardiovascular Outcomes Research With PCSK9 Inhibition in Subjects with Elevated Risk (FOURIER) trial, which demonstrated that lowering of LDL-C with Evolocumab reduced the risk of major adverse limb events [13]. Our result extends the FOURIER finding by further suggesting that LDL-C modulating therapies may decrease lifetime burden of PAD. In fact, there are few studies which evaluate the impact of genetic LDL-C modulation on PAD. A 2009 study of 13,634 EUR individuals demonstrated a significant reduction in risk for prevalent PAD, which did not extend to incident disease [3]. We again confirm this finding in White individuals. Results were not significant for Black or Hispanic individuals, perhaps due to lower power to detect associations in these populations.

We found a significant reduction in the risk of all-cause dementia amongst individuals with genetically reduced *PCSK9* expression. Both vascular dementia and non-vascular dementias, including Alzheimer Disease, have a recognized vascular component underlying their pathophysiology [14]. In the recent genetic meta-analysis of Alzheimer’s disease, several genes were implicated in lipid processing, including *APOM*, *APOA5*, and *ABCA1* [15]. Elevated LDL-C increases the risk of both vascular dementia and Alzheimer disease, although treatment with statins has not been shown to consistently reduce the risk of dementia [16]. Our findings add to the literature by providing evidence of a protective effect of LDL-C modulation on the risk of dementia. This hypothesis could be confirmed in additional observational studies and tested in future clinical trials.

We did not replicate the increased risk of diabetes seen in other studies examining pleiotropy of PCSK9 LoF. One possible explanation is heterogeneity in the definitions used for T2D. Whereas several previous studies used a diabetes definition involving laboratory data, ours utilized only ICD codes, allowing for the possibility of case-control misclassification. Further, our study had less power to detect a difference in T2D risk when compared to other larger published meta-analyses [4]. These considerations aside, our study is consistent with results from FOURIER trial subgroup analysis, which demonstrated no difference in risk for new onset diabetes in patients treated with Evolocumab [17].

The addition of statin use as a covariate diminished the effect estimates and strength of association for all atherosclerotic phenotypes. For dementia, the effect estimate was reversed, though the strength of association no longer met our significance threshold. These results highlight the benefits of pharmacologic control of lipids in individuals with higher baseline LDL-C, with HMG CoA reductase inhibition flattening the risk profile between those with and without genetic PCSK9 LoF. Notably, in spite of controlling for statin use, individuals with genetic PCSK9 LoF had further reduction in risk from both CAD and AAA, arguing for an additional benefit of PCSK9 inhibition in modifying risk of atherosclerotic vascular disease in individuals with suboptimal lipid control who are already taking a statin.

Limitations of our work include that the MVP cohort is predominantly male, with reduced power to examine for differences in females. Further, it is possible that pleiotropy could impact our results due to the high phenotypic correlations among atherosclerotic vascular diseases [12]. Additional observational and intervention studies are warranted to more definitely establish causality. Finally, our GRS was established in White population. SNPs included in the score had varying MAFs by genetic ancestry and while results are scaled by the ancestry specific GRS effect on LDL-C, care should be taken in interpreting results in Black and Hispanic populations.

In summary, we generated a GRS predicting *PCSK9* function and demonstrated a reduction in risk of several important extra-coronary atherosclerotic phenotypes in addition to known effects on CAD, including PAD, AAA, and ischemic stroke. We also highlight a novel reduction in risk of dementia, supporting a well-recognized vascular component to cognitive impairment. Our work emphasizes the benefits of biobank-driven genetics research and highlights the power of the MVP, a unique and diverse biobank of US veterans.

## Supporting information

S1 File (XLSX)

S1 Data (DOCX)
